# Annexin V-induced rat Leydig cell proliferation involves Ect2 via RhoA/ROCK signaling pathway

**DOI:** 10.1038/srep09437

**Published:** 2015-03-24

**Authors:** Jun Jing, Li Chen, Hai-Yan Fu, Kai Fan, Qi Yao, Yi-Feng Ge, Jin-Chun Lu, Bing Yao

**Affiliations:** 1 Center of Reproductive Medicine, Nanjing Jinling Hospital, Nanjing University School of Medicine, Nanjing 210002, China

## Abstract

This study investigated the effect of annexin V on the proliferation of primary rat Leydig cells and the potential mechanism. Our results showed that annexin V promoted rat Leydig cell proliferation and cell cycle progression in a dose- and time-dependent manner. Increased level of annexin V also enhanced Ect2 protein expression. However, siRNA knockdown of Ect2 attenuated annexin V-induced proliferation of rat Leydig cells. Taken together, these data suggest that increased level of annexin V induced rat Leydig cell proliferation and cell cycle progression via Ect2. Since RhoA activity was increased following Ect2 activation, we further investigated whether Ect2 was involved in annexin V-induced proliferation via the RhoA/ROCK pathway, and the results showed that annexin V increased RhoA activity too, and this effect was abolished by the knockdown of Ect2. Moreover, inhibition of the RhoA/ROCK pathway by a ROCK inhibitor, Y27632, also attenuated annexin V-induced proliferation and cell cycle progression. We thus conclude that Ect2 is involved in annexin V-induced rat Leydig cell proliferation through the RhoA/ROCK pathway.

Annexin V is a member of the annexin family of proteins, which consist of 12 annexins in mammals^1^. Common features among the annexins include structural similarities and calcium-dependent phospholipid binding. Annexin V was first identified as a protein with structural similarity to annexin I, a mediator of the anti-inflammatory activity of glucocorticoids via inhibition of phospholipase A2^2^,^3^ and an inhibitor of blood coagulation^4^. Annexin V also shows protein kinase C-inhibitory activity^5^, and it forms calcium channels on phospholipid membranes^6^. Although the biochemical properties suggest important roles of annexin family proteins in cell functions, the function of these proteins in physiological process is still obscure^7^. It is synthesized in the anterior pituitary gland^8^ and produced by pituitary gonadotropes under the regulation of gonadotropin releasing hormone (GnRH)^9^,^10^,^11^,^12^. Kawaminami et al. found that annexin V was expressed in the regressing corpus luteum^13^. The expression of annexin V in luteal cells was accompanied by apoptotic change that was inhibited by local administration of a GnRH receptor antagonist, thus annexin V synthesis is stimulated by GnRH in the ovary.

Leydig cells are the main source of steroid hormones in the mammalian testis, preserving the normal functions of which determines the reproductive capacity and fertility of males. Testosterone is primarily produced by adult Leydig cells and is important to maintain spermatogenesis and secondary sexual characteristics. It has been reported that annexin V is expressed by Leydig and Sertoli cells in rat^14^,^15^. Annexin V and 3-β-hydroxysteroid dehydrogenase (HSD) were also shown to colocalize in the Leydig cells^16^. Our previous studies showed that GnRH agonist increased the mRNA and protein levels of annexin V in primary rat Leydig cells *in vitro*^17^, as well as the secretion of testosterone^18^, annexin V could increase testosterone production and promote cell proliferation of Leydig cells. During the puberty, the proliferation of Leydig cell precursors is required to increase the number of Leydig cells. In this study, we found the existence of functional relationship between annexin V and Leydig cell proliferation. With the method of differential proteomics, a total of 33 proteins were identified as differentially expressed in annexin V-treated Leydig cells^19^. Among these proteins, Ect2 was correlated with Leydig cell proliferation and chosen for further study. The upregulation of Ect2 implied a subtle cell proliferation trend induced by annexin V.

Based on these data, we hypothesize that upregulation of Ect2 contributes to rat Leydig cell proliferation induced by exogenous annexin V. Thus the present study aimed to examine the effects of annexin V on Ect2 expression and its potential link with the proliferation of rat Leydig cells. The potential mechanisms of Leydig cell proliferation may offer new opportunities for treating testosterone deficiency.

## Results

### Annexin V promotes rat Leydig cell proliferation and cell cycle progression

**Figure 1:**
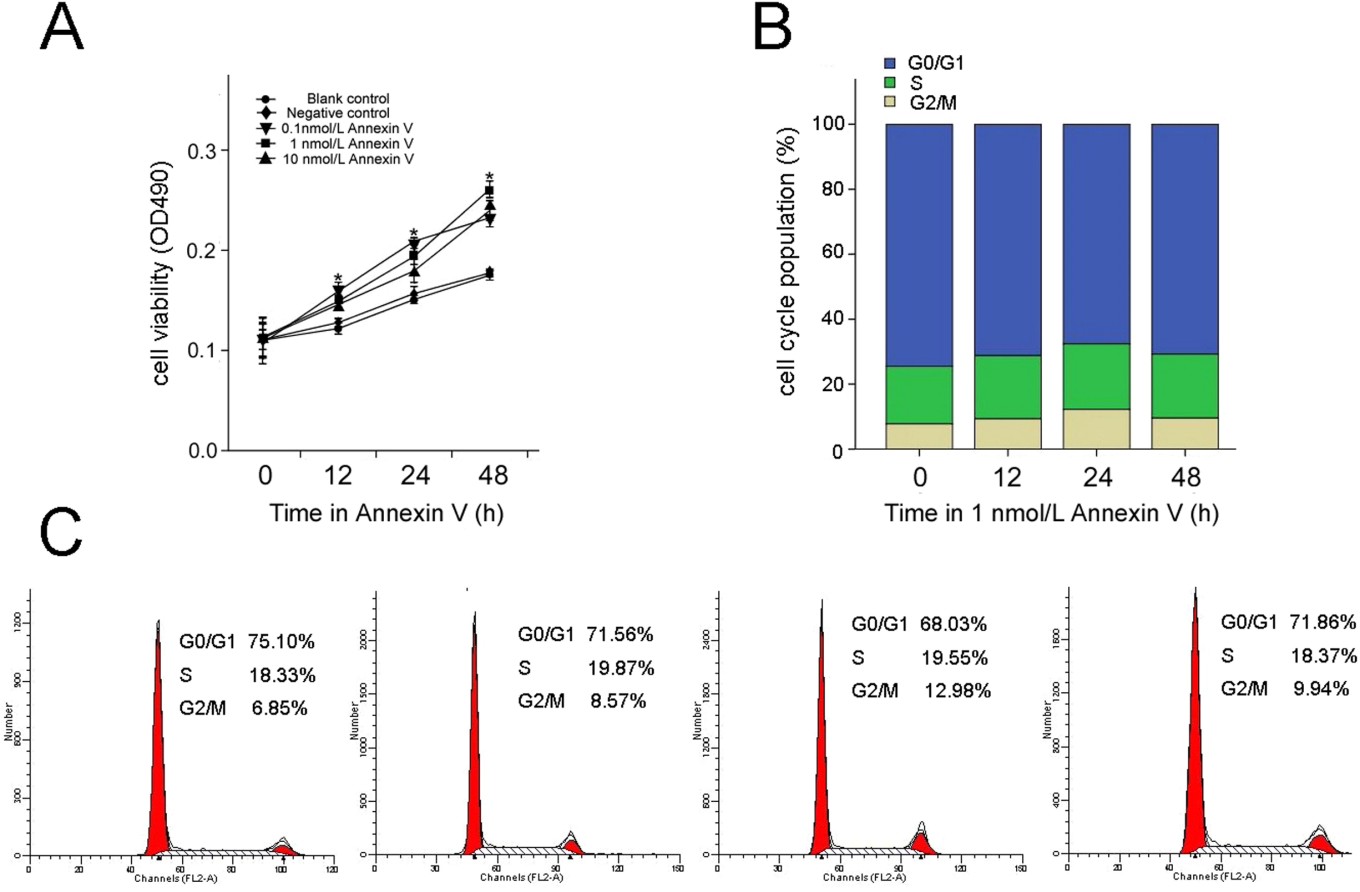
The influence of annexin V on rat Leydig cell proliferation and cell cycle progression. (A) Cell proliferation was increased after treated with different concentrations of annexin V in comparison with blank control and negative control, as determined by the MTT assay; the results are expressed as the mean of cell proliferation viability (*n* = 5). (B and C) Cell cycle was confirmed after treated with 1 nmol/L annexin V for different time (12, 24, 48 h) in comparison with 0 h, as determined by flow cytometry; the results are expressed as the mean of G2/M cell population (*n* = 3). Asterisks indicate the statistical significance (*P < 0.01), *vs.* Blank control.

Proliferation viability of rat Leydig cells exposed to different concentrations of annexin V for 0–48 h was assessed by MTT assay (Fig. 1A). No differences were observed in Leydig cell viability between the negative control and the blank control groups (*P* > 0.05; *n* = 5) in different time periods. However, the cell viability increased compared to the blank control group when Leydig cells were treated with 0.1–10 nmol/L annexin V for 12–48 h (*P* < 0.01; *n* = 5). Significant increase in proliferation viability occurred under 1 nmol/L annexin V at 48 h. Compared with the blank control, the cell proliferation viability was increased by 49% under 1 nmol/L annexin V treatment (*P* < 0.01; *n* = 5).

The effect of annexin V on the cell cycle of rat leydig cells was evaluated using flow cytometry, and the obtained results are illustrated in Fig. 1B and C. After 24 and 48 h exposure to 1 nmol/L annexin V, the G0/G1 cell population decreased (*P* < 0.01; *n* = 3) compared with the control group, while the G2/M cell population significantly increased (*P* < 0.01; *n* = 3). Although the differences were not significant, compared with the control group (*P* < 0.05; *n* = 3) the G2/M cell population increased after 1 nmol/L annexin V treatment for 12 h. For the S phase population, there was an insignificant but numerical increase (*P* > 0.05; *n* = 3) under 1 nmol/L annexin V treatment from 12 to 48 h.

### Annexin V increases the protein expression levels of Ect2

**Figure 2:**
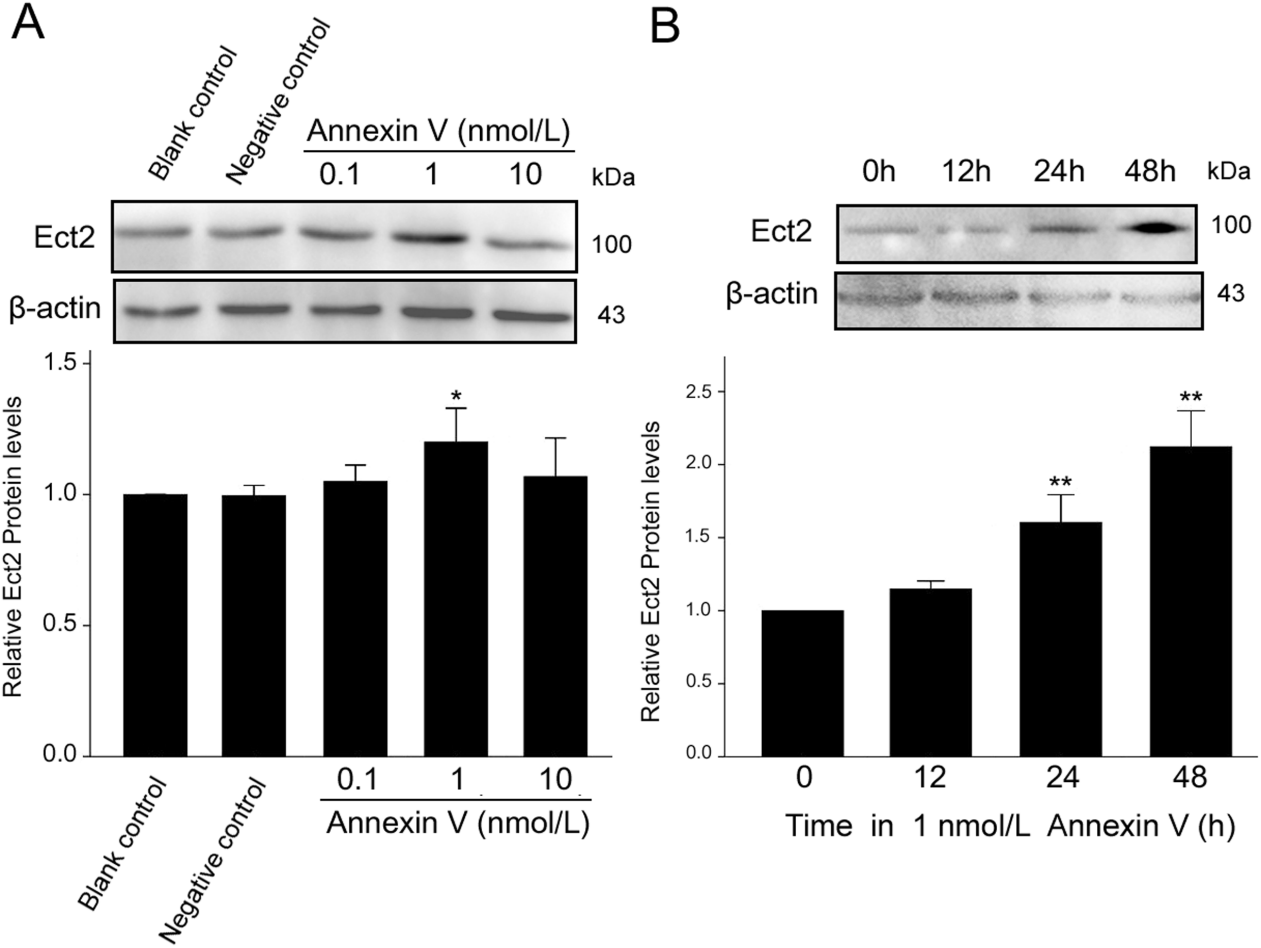
The influence of annexin V on Ect2 protein expression in rat Leydig cells. The cells were treated with annexin V in comparison with blank control and negative control, and the protein expression of Ect2 was detected by Western blotting. Annexin V increased Ect2 protein levels in a dose- (A) and time-dependent (B) manner (*n* = 3). Asterisks indicate the statistical significance (*P < 0.05, **P < 0.01), *vs.* Blank control.

To investigate the mechanism of annexin V affecting Leydig cell proliferation, we used Western blot to detect the change of Ect2 protein level. The effects of annexin V on Ect2 expression were in a dose-dependent manner (Fig. 2A). Significant increase in Ect2 protein expression occurred under 1 nmol/L annexin V. Compared with the blank control, the protein expression of Ect2 was increased by 18% under 1 nmol/L annexin V treatment (*P* < 0.05; *n* = 3). However, no differences were observed in the protein expression of Ect2 between the negative control and blank control (*P* > 0.05; *n* = 3). Annexin V (1 nmol/L) also displayed a time-dependent effect on Ect2 expression, as shown in Fig. 2B. The protein expression of Ect2 was increased by 61% and 113% under 1 nmol/L annexin V at 24 and 48 h respectively (*P* < 0.01; *n* = 3). However, there was a trend of insignificant increase (*P* > 0.05; *n* = 3) under 1 nmol/L annexin V treatment at 12 h.

### Blockage of Ect2 activity attenuates rat Leydig cell proliferation

**Figure 3:**
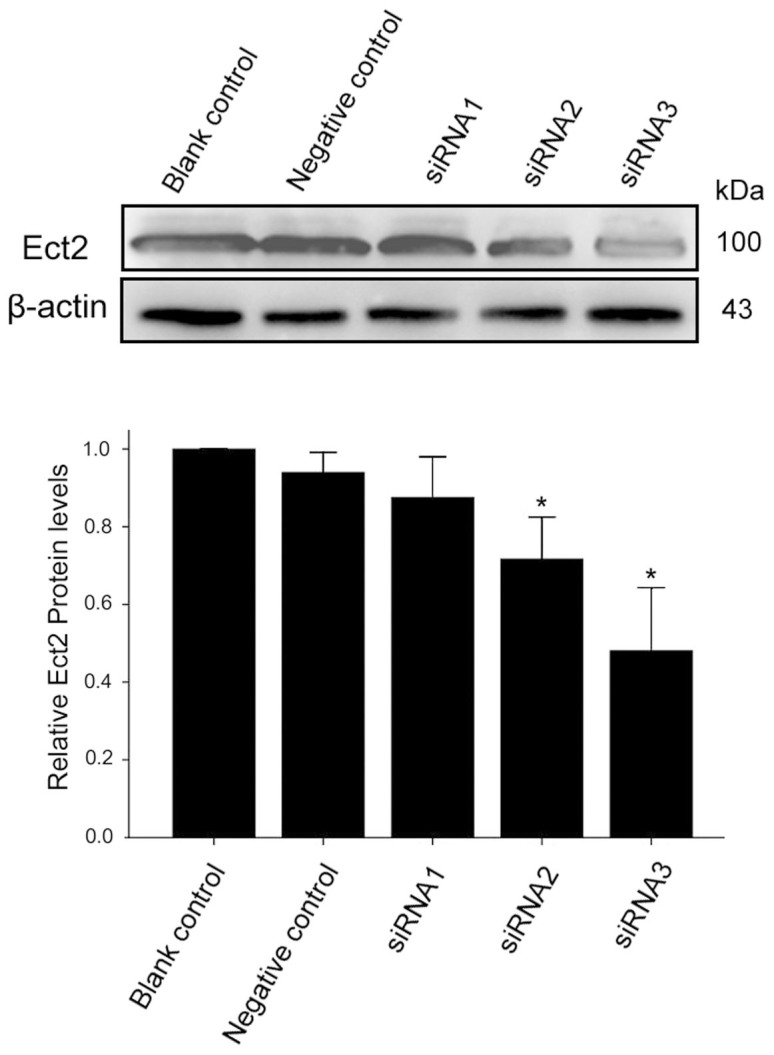
The inhibitory effect of siRNA duplexes on Ect2 expression in rat Leydig cells. The cells were transfected with 3 different siRNAs (100 nmol/L) in comparison with blank control and negative control (scrambled siRNA) for 48 h, and the protein expression of Ect2 was detected by Western blotting (*n* = 3). The protein expression of Ect2 decreased significantly by 24% and 49%, respectively, for siRNA2 and siRNA3. * *P* < 0.05, compared with Negative control.

To examine the silencing effect of siRNAs-Ect2 on Ect2 protein expression, we transfected the 3 siRNA duplexes and the scrambled siRNA into Leydig cells respectively. After transfection for 48 h, the cells were collected for Western blotting. Compared with the blank control, 2 duplexes showed an inhibitory effect on Ect2 expression, and the protein expression of Ect2 decreased by 24% and 49% respectively (*P* < 0.01; *n* = 3); siRNA-3 was the best among the 3 duplexes (Fig. 3).

**Figure 4:**
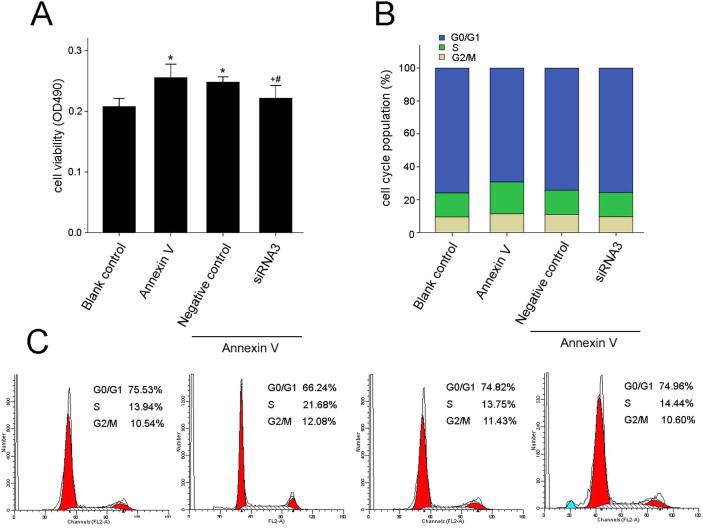
Annexin V-induced Leydig cell proliferation was attenuated by depletion of Ect2. The cells were transfected without or with siRNA3 or scrambled siRNA (negative control) followed by treatment with blank control or annexin V for 48 h. (A) The cell proliferation was determined using MTT assay; the results are expressed as cell proliferation viability (*n* = 5). (B and C) Cell cycle was confirmed using flow cytometry; the results are expressed as the G2/M cell population (*n* = 3). *P < 0.01 *vs* Blank control; +*P* < 0.01 *vs.* annexin V; and #*P* < 0.05 *vs.* annexin V+ Negative control.

To further explore the role of Ect2 in annexin V-induced Leydig cell proliferation, we employed siRNA to assess the effect of Ect2 depletion on annexin V-induced proliferation using MTT assay and flow cytometric analysis. The administration of 1 nmol/L annexin V for 48 h caused a significant increase in cell proliferation viability in contrast with control (*P* < 0.01; *n* = 5; Fig. 4A). Flow cytometry analysis further indicated that the G2/M cell population was significantly increased in annexin V group in contrast with the control group (Fig. 4B and C). However, the increase in both the cell proliferation viability and the G2/M cell population induced by annexin V was significantly inhibited by transfection with Ect2-siRNA (Fig. 4), compared with non-transfected cells and cells transfected with scrambled siRNA, suggesting the involvement of Ect2 in annexin V-induced proliferation.

### Ect2 participates in annexin V-induced Leydig cell proliferation via RhoA/ROCK signaling pathway

**Figure 5:**
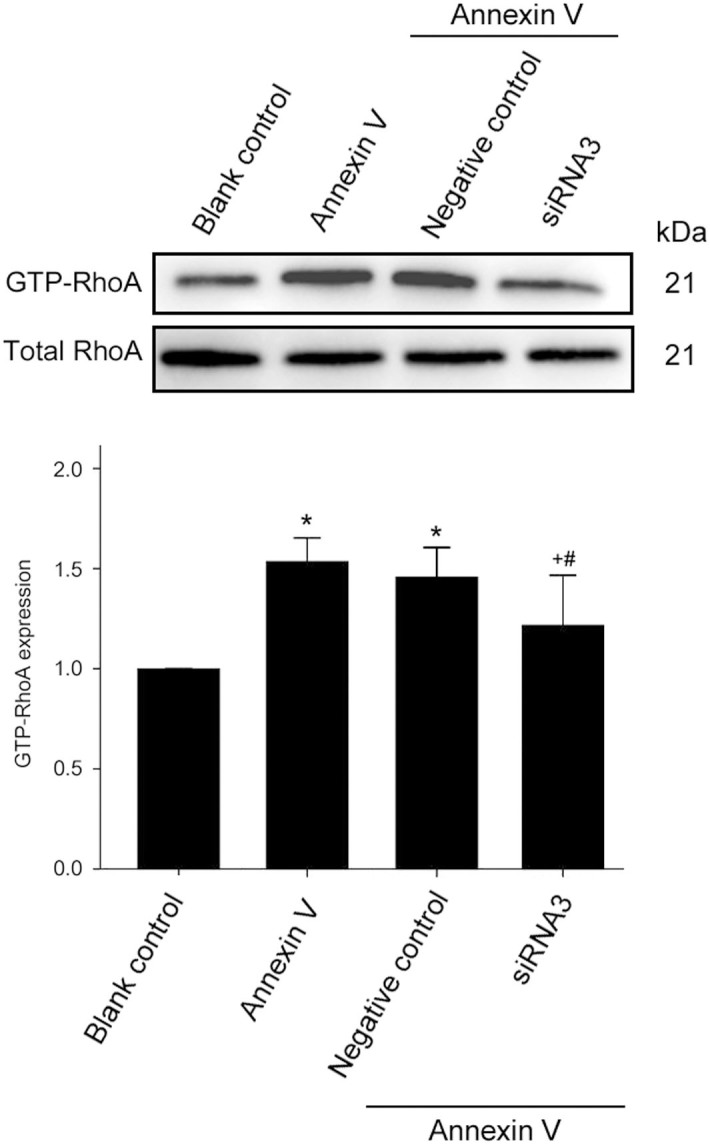
Knockdown of Ect2 expression blocked annexin V-induced increase in RhoA activity. The cells were transfected without or with siRNA3 or scrambled siRNA (negative control), and the cells were then treated with blank control or annexin V for 48 h. RhoA activity was measured using a pull-down assay as described in Methods (*n* = 3). *P < 0.01 *vs.* Blank control; +*P* < 0.01 *vs.* annexin V; and #*P* < 0.05 *vs.* annexin V+ Negative control.

**Figure 6:**
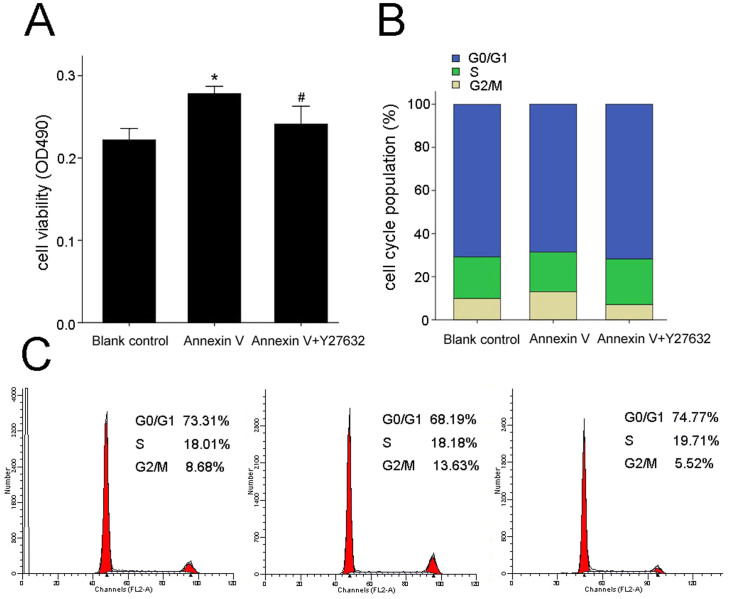
Inhibition of ROCK attenuated annexin V-induced Leydig cell proliferation. Following pretreatment of cells with 10 μmol/L Y27632 (a selective inhibitor of ROCK) for 30 min, then treated with blank control or annexin V for 48 h. The MTT assay (A) and flow cytometry (B and C) were used to identify proliferation cells (*n* = 3). *P < 0.01 *vs.* Blank control; #*P* < 0.05 *vs.* annexin V.

RhoA activity was measured by an affinity pull-down assay using the GST fusion protein rhotekin that only recognizes the active form of RhoA (GTP-RhoA). Treatment with 1 nmol/L annexin V for 48 h caused a significant increase in RhoA activity in contrast with control (*P* < 0.01; *n* = 3; Fig. 5). This effect may contribute to annexin V-induced Leydig cell proliferation, because transfection with Ect2-siRNA attenuated annexin V-induced increase in RhoA activity (Fig. 5), compared with non-transfected cells or cells transfected with the scrambled siRNA (*P* < 0.01; *n* = 3). If the RhoA/ROCK pathway activation promotes Ect2-dependent G2/M cell population increase in annexin V-induced Leydig cell proliferation, inhibition of this down-stream pathway should attenuate the effect of annexin V. We thus measured the effect of Y27632, a selective ROCK inhibitor, on the annexin V-induced proliferation and found that the increase in both the cell proliferation viability and the G2/M cell population induced by annexin V was significantly inhibited by the concurrent administration of Y27632 (10 μmol/L; Fig. 6), indicating the involvement of RhoA/ROCK pathway in Ect2-dependent G2/M cell population increase during annexin V-induced Leydig cells proliferation.

## Discussion

This study revealed that annexin V promoted rat Leydig cell proliferation in a dose- and time-dependent manner. Meanwhile, flow cytometry showed a significant increase of cells in the G2/M phase. Cell proliferation viability was related to the sum of cells in S and G2/M phase of cell cycle, so increasing the transition from the G2 phase to M phase promoted cell proliferation viability of rat Leydig cells. We also demonstrated that Ect2 was involved in annexin V-induced proliferation of rat Leydig cells. Ect2, the epithelial cell transforming sequence 2 oncogene, was found in 1993 by Miki et al. in epithelial cells, which is located on chromosome 3q26^20^. The C-terminal of the Ect2 protein catalyzes guanine nucleotide exchange on the Rho family of small GTPases, while the N-terminal contains a domain related to cell cycle regulator/checkpoint control protein^21^. The expression of this gene is elevated with the onset of DNA synthesis and remains elevated during G2 and M phases^22^. In situ hybridization analysis showed that Ect2 was highly expressed in mitotic cells during liver regeneration^23^. These results suggest that Ect2 is an important player in cell cycle machinery involved in the regulation of cell division.

We also found that Ect2 knockdown by siRNA attenuated the annexin V-induced rat Leydig cell proliferation. A previous study reported that microinjection of affinity-purified anti-Ect2 antibody into interphase cells also inhibited cytokinesis^24^, while depletion of Ect2 by siRNA oligonucleotides reduced the rate of glioblastoma cell proliferation too^25^.

Taken together, these findings suggest that there is a common mechanism by which Ect2-dependent G2/M cell population increase contributes to the annexin V-induced cell proliferation. Ect2, a guanine nucleotide exchange factor (GEF) that activates RhoA during cytokinesis, is regulated by phosphorylation and subcellular localization^26^. Expression of the dominant negative form of Ect2 completely suppressed both the rise of GTP-Rho in the telophase and the increased GDP-GTP exchange activity in the mitotic cell extracts^27^. Ect2 is normally in an inactive conformation, partially due to the intramolecular interaction between the BRCT domains and the C-terminal domain of Ect2, which blocks its catalytic activity for guanine nucleotide exchange toward RhoA^28^. This impact on Rho signaling was accompanied by reduced junctional localization of Ect2 and the centralspindlin complex containing Ect2^29^. A previous study found that Ect2 first becomes active in prophase during its translocation from the nucleus into the cytoplasm, activating RhoA to induce the formation of metaphase cortex^30^. The PH domain is required for the cortical localization of Ect2, but its molecular function is not known. In cultured human cells, the PH domain was observed to interact with anillin, a contractile ring protein that scaffolds actin and myosin and interacts with RhoA^31^. As one of at least 25 RhoGEFs that can activate the RhoA small GTPase, Ect2 was demonstrated in cell culture studies with established cell lines as essential for mammalian cell cytokinesis and proliferation^32^. Using primary cells, a previous study showed that guanine exchange factors GEF-H1 and Ect2 must be downregulated for megakaryocyte polyploidization^33^. The Rho GTPase-activating protein (GAP) domain of CYK-4 was shown to promote the activation of RhoA during cytokinesis^34^. Both the guanine nucleotide exchange function and the membrane targeting of Ect2 are essential for RhoA activation and cleavage furrow formation in human cells^35^. Ect2 has been demonstrated to subject to phosphorylation/dephosphorylation throughout the meiosis of oocytes, and PBI emission is temporally associated with Ect2 dephosphorylation too^36^. These effects were associated with the inhibition of mitogen-induced activation of the MAPK pathway and the suppression of anillin, Ect2, and cyclin B1 among other proteins involved in mitosis, which would be expected to reduce the activation of endogenous RhoA at the cell equator^37^.

Consistent with these results, we reported that annexin V treatment also evoked a marked activation of RhoA, which was abolished by the knockdown of Ect2 using siRNA, suggesting that RhoA is a downstream effector of Ect2 activation. However, the activation mechanism of RhoA signaling has not yet been elucidated. Our results indicated that annexin V-induced RhoA activation may account for Ect2-dependent increase of G2/M cell population. It is important to note that our data do not exclude the possibility that the effect of Ect2 on annexin V-induced Leydig cell proliferation may be mediated, at least in part, by RhoA/ROCK-independent pathways. For example, Ect2 had an intrinsically distinct GTPase specificity profile in the nucleus versus the cytoplasm, while nuclear Ect2 bound preferentially to Rac1, cytoplasmic Ect2 bound to RhoA^38^.

## Methods

### Reagents

Recombinant rat annexin V was synthesized in our laboratory^39^. The full-length encoding sequence of rat annexin V was chemically synthesized and inserted into the HIS fusion expression vector pET28a. The expression of the fusion protein HIS-annexin V (36 kDa) was induced by isopropyl-beta-D-thiogalactoside (IPTG) under the control of the T7 promoter, and the products were purified by affinity chromatography. The protein annexin V can be efficiently expressed in E. coli. The biological anticoagulation activity of annexin V was determined by the modified activated partial thromboplastin time (APTT) test, which confirmed that the synthesized annexin V has strong anticoagulant activity. Dulbecco's modified Eagle's medium (DMEM)/Ham's nutrient mixture F12 (DMEM/F12) was purchased from Invitrogen (Grand Island, NY, USA). Percoll, HEPES, collagenase type I were from Sigma-Aldrich Corporation (St Louis, MO, USA). The 3-(4, 5-dimethylthiazol-2yl)-2,5-diphenyl tetrazolium bromide (MTT) was obtained from Amresco Inc. (Solon, OH, USA). The Cell Cycle Detection Kit was obtained from Biovision (USA). Y27632 was purchased from Tocris (Bristol, UK). Rabbit anti-Ect2 polyclonal antibody and rabbit anti-annexin V polyclonal antibody was from Santa Cruz (CA, USA). Rabbit anti-RhoA polyclonal antibody, rabbit anti-β-actin antibody, and goat anti-rabbit secondary antibodies were purchased from Cell Signaling (USA).

### Leydig cell isolation and culture

Male Sprague-Dawley rats (9–10 weeks old) were bred in our laboratory. The animal room was maintained at 22–24°C under a constant 12 h light:12 h darkness cycle. Animals were fed with standard pellet diet and water *ad libitum*. The procedure of animal experiments was performed following the guidelines for animal treatment of Nanjing Jinling Hospital and approved by the local ethics committee, which is in accordance with the principles and procedure of the NIH guide for the care and use of laboratory animals.

Leydig cells were prepared from the immature rat testes by collagenase treatment as described previously^40^. Briefly, decapsulated testes were incubated with collagenase (0.25 mg/mL) for 20 min at 37°C. The crude interstitial cells were collected by centrifugation at 1,000 × g for 10 min, and then washed twice with HBSS containing 0.1% (w/v) BSA. To obtain pure Leydig cells, the crude cell suspension was loaded on top of a discontinuous Percoll gradient (20, 40, 60, and 90% Percoll in HBSS) and subsequently centrifuged at 800 × g for 20 min. The fractions enriched in Leydig cells were obtained and centrifuged in a continuous, self-generating density gradient starting with 60% Percoll at 20,000 × g for 30 min at 4°C.

The total number of cells and the percentage of 3-β-HSD-positive cells were determined with this Leydig cell preparation^41^. The purity of the Leydig cells was 85–90%. The cell viability, as assessed by Trypan blue exclusion, was greater than 90%. The purified Leydig cells were washed twice with DMEM-F/12 and resuspended in DMEM-F/12 supplemented with 15 mmol/L HEPES (pH 7.4), 1 mg/mL BSA, 365 mg/L glutamine, 100 IU/mL penicillin, and 100 μg/mL streptomycin.

For culturing, 2 mL cell suspension containing 10^6^ cells/mL were placed into each well of 6-well plate (Costar, NY, USA) and incubated at 34°C in a humidified atmosphere of 5% CO_2_-95% air. Annexin V (1 nmol/L) was preincubated with sufficient (10 nmol/L) annexin V specific antibody for 1 h at 37°C, which would inactivated annexin V function and served as negative control. The cells were incubated with fresh medium containing increasing concentrations of annexin V (0.1, 1, and 10 nmol/L, respectively) or with blank control or negative control for different time (0, 12, 24, and 48 h, respectively).

### Transfection of siRNAs in Leydig cells

Three siRNA duplexes were synthesized commercially by Invitrogen with the help of tools available online (cnservice.invitrogen.com). The sequences were as follows: siRNA1, 5′-GGU GUC UAC UUC AAA UGU U(dTdT)-3′; siRNA2, 5′-GCA AGG AGG UAC CUA UUU A(dTdT)-3′; and siRNA3, 5′-GCA GAU GCU GAG AAU CUU A(dTdT)-3′. They were designed to target different coding regions of the rat Ect2 mRNA sequence (Gene Bank Accession NO. NM_001108547.2).

The day before transfection, 2 × 10^6^ cells in 2 mL of DMEM without antibiotics were plated so that cells will be 70–90% confluent at the time of transfection. In the following day, the cells were transfected with siRNA in 50 μL PBS at a final concentration of 100 nmol/L. After mixing gently and incubating for 1 min, the diluted GenEscortTM II at a final ratio of GenEscortTM II (μL) to siRNA (μg) of 2:1 was added, mixed gently, and then incubated for 10–15 min at room temperature to allow transfection complex formation. After 48 h at 34°C with 5% CO_2_, the transgene expression was tested. A control and a negative control were also contained in the 6-well plates.

### Cell viability detection by MTT assay

The MTT [3-(4,5-dimethyl-2-thiazolyl)-2,5-diphenyl-2-H-tetrazolium bromide] assay is a colorimetric assay for assessing cell proliferation. Briefly, cells were plated onto a 96-well culture plate at 10^4^ cells/well in 100 μL of culture medium. After the incubation, 20 μL of MTT solution (5 mg/mL) were added into each well followed by further incubation for 4 h. The supernatants were removed, and 150 μL of DMSO were added into the wells to dissolve the formazan crystal. After 10 min of incubation at room temperature, the absorbance was measured on an automated microplate reader (Bio-Rad, Japan) at 490 nm.

### Cell cycle assay by flow cytometry

Cells were harvested in cold PBS, fixed in 70% alcohol, and stored at 4°C. The fixed cells were washed with PBS once and suspended in 500 μL of PI staining solution containing 100 μg/mL RNAse A, and were then incubated in the dark for 30 min. The cell cycle was measured by a Becton Dickinson FACS analysis system, and the quantitation of cell cycle distribution was carried out using Multicycle Software.

### SDS-PAGE and Western blot analysis

Cells were washed twice with ice-cold PBS and lysed in 200 μL of ice-cold RIPA buffer containing 150 mmol/L NaCl, 1% Nonidet P-40, 0.25% deoxycholate, 0.1% sodium dodecyl sulfate (SDS), 50 mmol/L Tris (pH 7.4), 1 mmol/L phenylmethylsulfonyl fluoride (PMSF), 1 mmol/L Na_3_VO_4_, and 1 mmol/L NaF. The cell lysate was harvested and centrifuged at 10,000 × g and 4°C for 20 min. The supernatants were collected to new tubes, and the protein concentration of supernatants was determined using Bradford method. The total protein (30 μg) was then mixed with 6× loading buffer and boiled for 5 min. The sample mixture was run on 15% SDS-PAGE gels in 1× running buffer at 25 mA for 2 h. The protein was transferred to polyvinylidene difluoride membranes (PVDF; Millipore Corporation) at 70 V for 3.5 h in transfer buffer. Then, the membrane was blocked in TBST (0.5% Tween 20 in Tris-buffered saline) containing 5% (w/v) non-fat dry milk at 37°C for 1.5 h, and washed 3 times with TBST for 30 min. The membranes were incubated with respective primary antibodies for 16–18 h at 4°C. After washing, the membrane was incubated with horseradish peroxidase-conjugated secondary antibody, and the bands were visualized with ECL (Promega) as described by the manufacturer. The intensity of bands was quantitated by Quantity-One software.

### RhoA activation assay

RhoA activity was measured using a Rho activation assay kit (Cytoskeleton) according to manufacturer's instructions. The cells were lysed in cell lysis buffer and cleared by centrifugation. The supernatants were incubated with Rhotekin-Rho binding domain (RBD) glutathione affinity beads, which specifically bind to GTP-bound RhoA. The beads were washed, and the immunoprecipitated complex was resuspended in 2× Laemmli sample buffer and subjected to 15% SDS–PAGE, followed by Western blot analysis. Total RhoA protein was determined in separate Western blots and used to normalize GTP-bound RhoA densitometricunits.

### Statistical analysis

All data are expressed as mean ± standard deviation. Standard deviation was illustrated by bars in Figures. The differences between the means were analyzed by one-way analysis of variance (ANOVA) and the least significance difference (LSD) method. *P* values less than 0.05 were considered to be statistical significance.

## Supplementary Material

Supplementary Information Full-length blots
