# Supplementary material for: Annexin V-induced rat Leydig cell proliferation involves Ect2 via RhoA/ROCK signaling pathway
Source: Sci Rep. 2015 Mar 24;5:9437. doi: 10.1038/srep09437 (PMC5380157; doi:10.1038/srep09437)
Supplement: Supplementary Information — Full-length blots [file srep09437-s1.doc]

**Supplementary Information**

**Annexin V-induced rat Leydig cell proliferation involves Ect2 via RhoA/ROCK signaling pathway**

Jun Jing *****, Li Chen*****, Hai-Yan Fu, Kai Fan, Qi Yao, Yi-Feng Ge, Jin-Chun Lu, Bing Yao^#^

Center of Reproductive Medicine, Nanjing Jinling Hospital, Nanjing University School of Medicine, Nanjing 210002, China

***These authors contributed equally to this work**

^#^**Corresponding author:**

Dr. Bing Yao

Center of Reproductive Medicine, Nanjing Jinling Hospital, Nanjing University School of Medicine, Nanjing 210002, China

Phone: +86-25-8086-0174; Fax: +86-25-8086-0174

Email: [2424572228@qq.com](mailto:2424572228@qq.com)

**Supplementary Figures**

Note: The gels had been run under the same experimental conditions.  In addition, the gels had been directly transferred to PVDF membranes after running,  so the gel figures had not been taken. We need to pay more attention to our work in the future.

**Original Figures**

**
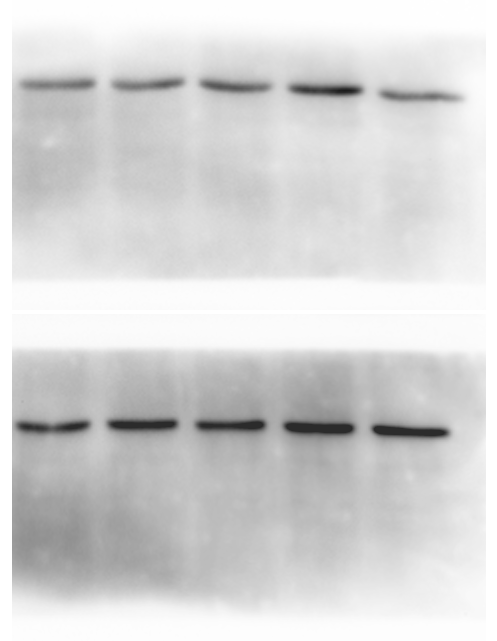

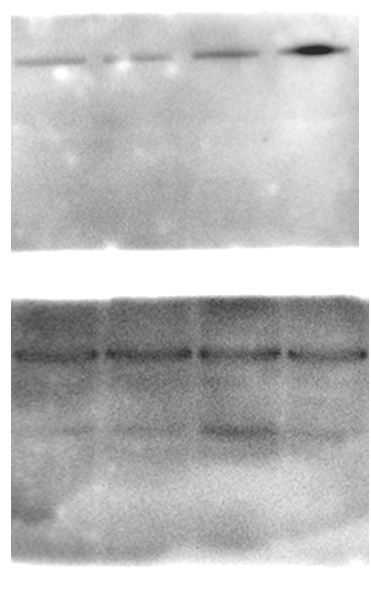
**

**Cutting Figures**

**
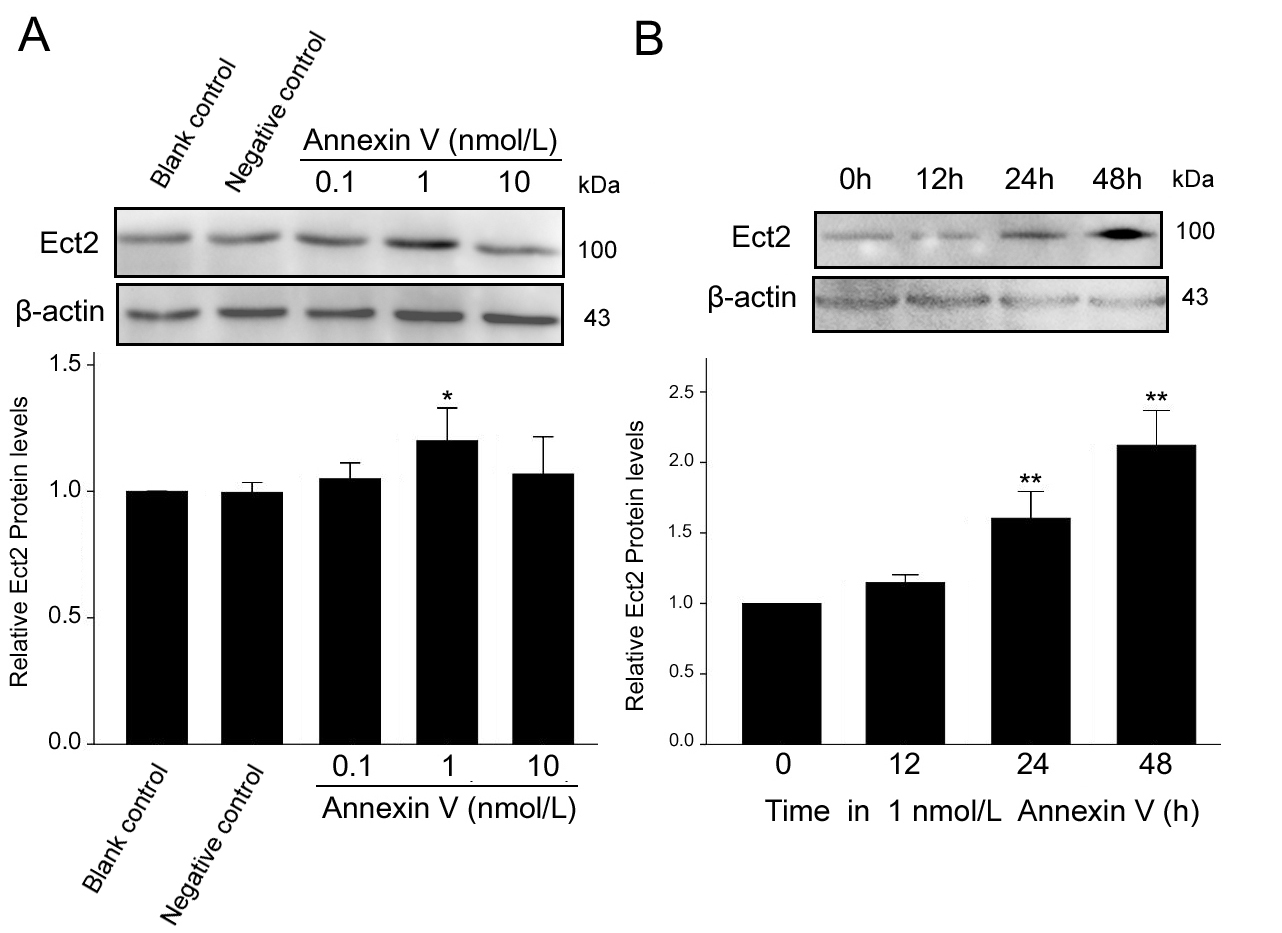
**

**Fig. 2.** **The influence of annexin V on Ect2 protein expression in rat Leydig cells.** Cell were treated with annexin V in comparison with blank control and negative control, and the protein expression of Ect2 was detected by Western blotting. Annexin V increased Ect2 protein levels in a dose- (A) and time-dependent (B) manner (*n* = 3). Asterisks indicate the statistical significance (**P* < 0.05, ***P* < 0.01), *vs.* Blank control.

**Original Figures Cutting Figures**


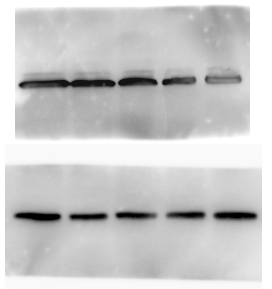

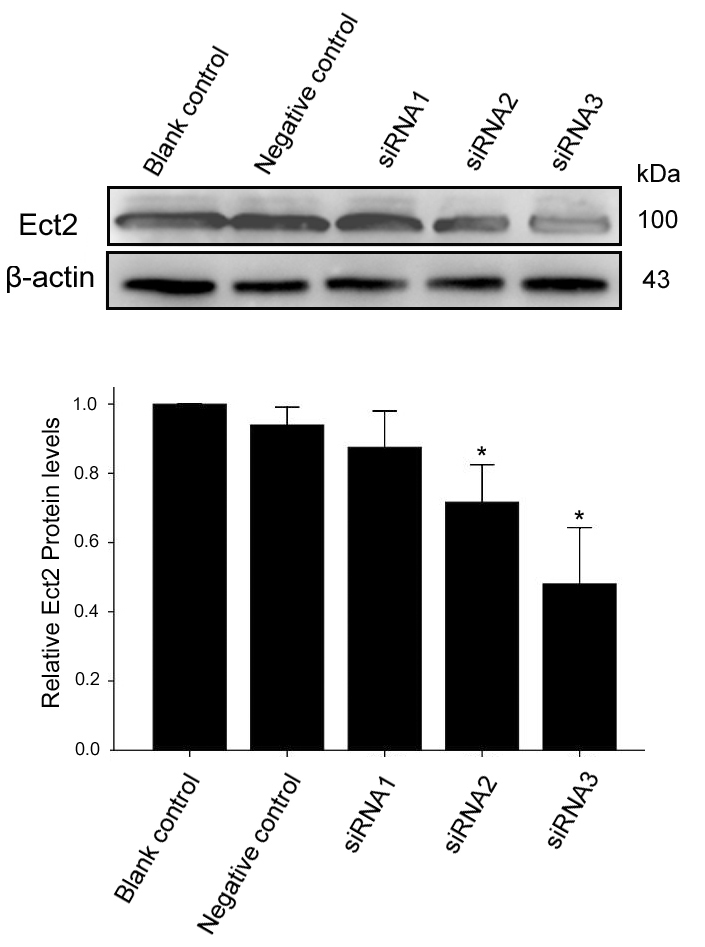


**Fig. 3.** **The inhibitory effect of siRNA duplexes on Ect2 expression in rat Leydig cells.** The cells were transfected with 3 different siRNAs (100 nmol/L) in comparison with blank control and negative control (scrambled siRNA) for 48 h, and the protein expression of Ect2 was detected by Western blotting (*n* = 3). The protein expression of Ect2 decreased significantly by 24% and 49%, respectively, for siRNA2 and siRNA3. * *P* < 0.05, compared with Negative control.

**Original Figures Cutting Figures**


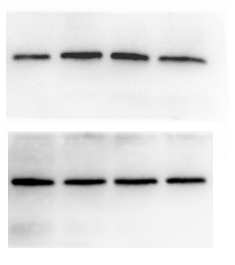

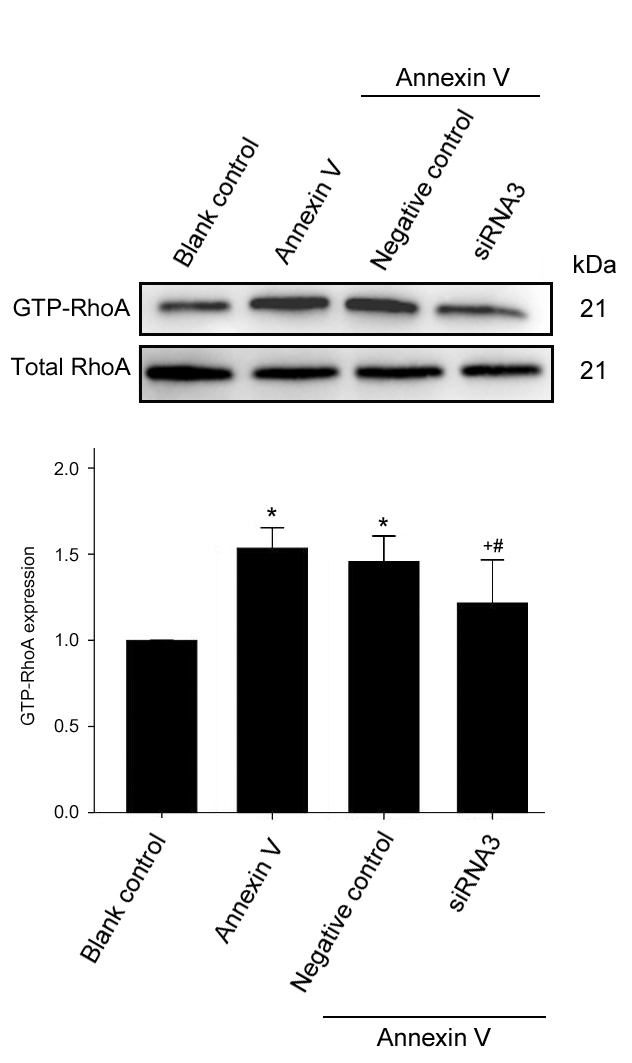


**Fig. 5.** **Knockdown of Ect2 expression blocked annexin-induced increase in RhoA activity.** The cells were transfected without or with siRNA3 or scrambled siRNA (negative control), and the cells were then treated with blank control or annexin V for 48 h. RhoA activity was measured using a pull-down assay as described in Material and Methods (*n* = 3). **P* < 0.01 *vs.* Blank control; +*P* < 0.01 *vs.* annexin V; and #*P* < 0.05 *vs.* annexin V+ Negative control.
